# Number of teeth lost on diet quality and glycemic control in patients with type 2 diabetes mellitus

**DOI:** 10.20945/2359-3997000000429

**Published:** 2022-01-14

**Authors:** Danieli Londero da Silveira, Laura Emanuelle da Rosa Carlos Monteiro, Christofer da Silva Christofoli, Beatriz D. Schaan, Gabriela Heiden Telo

**Affiliations:** 1 Universidade Federal do Rio Grande do Sul Porto Alegre RS Brasil Programa de Pós-graduação em Endocrinologia, Universidade Federal do Rio Grande do Sul, Porto Alegre, RS, Brasil; 2 Universidade Federal do Rio Grande do Sul Faculdade de Medicina Porto Alegre RS Brasil Faculdade de Medicina, Universidade Federal do Rio Grande do Sul, Porto Alegre, RS, Brasil; 3 Hospital de Clínicas de Porto Alegre Porto Alegre RS Brasil Hospital de Clínicas de Porto Alegre, Porto Alegre, RS, Brasil; 4 Pontifícia Universidade Católica do Rio Grande do Sul Porto Alegre RS Brasil Programa de Pós-graduação em Medicina e Ciências da Saúde, Pontifícia Universidade Católica do Rio Grande do Sul, Porto Alegre, RS, Brasil

**Keywords:** Diabetes mellitus, blood glucose, oral health, tooth loss

## Abstract

**Objectives:**

To describe the oral health profile and evaluate the impact of tooth loss on diet quality and glycemic control among 66 patients with type 2 diabetes (T2DM) treated in an endocrinology outpatient clinic at a teaching hospital.

**Materials and methods:**

Questionnaires about diabetes self-care (SDSCA), masticatory ability, diet quality, anxiety level about dental treatment, and oral health were applied. Laboratory tests were retrieved from medical records or newly collected samples.

**Results:**

The presence of fewer than 21 teeth was associated with an unsatisfactory self-perceived masticatory ability (r = 0.44; p = 0.007). Most participants reported not having received guidance on oral health from their endocrinologists (81.8%) and having had the last visit to the dentist 2 years or more before the study (36.8%). The mean HbA1c level in the group with fewer than 21 teeth was comparable to that in the group with functional dentition (8.9 ± 1.5 and 8.7 ± 1.6%, respectively; p = 0.60).

**Conclusion:**

Adults with T2DM have a high prevalence of tooth loss and lack of information about oral hygiene care. Our results reinforce the need for more effective communication between medical and dental care teams.

## INTRODUCTION

Achievement of glycemic control with the adoption of a healthy diet, along with maintenance of adequate body weight and control of serum lipids and blood pressure, is one of the main pillars of the management of type 2 diabetes mellitus (T2DM) (1).Robust evidence demonstrates that adherence to self-care in diabetes enables and enhances therapeutic success, mediating satisfactory results through a reduction in cardiovascular risk and improvement in metabolic control, quality of life, symptoms of anxiety, and depression (2).

The prioritization of consumption of foods that are fresh instead of those that are rich in fat, sodium, and sugar contributes to maintaining metabolic control (3). Poor oral health, represented by partial or total tooth loss, is associated with a higher probability of masticatory difficulty (4). One of the consequences of this complication is a preference for foods based on consistency (4), which in turn can compromise the individual’s nutritional status and general health, considering the low nutritional value of some of these foods (5).

A follow-up by a professional multidisciplinary team can help prevent these changes in eating patterns due to abnormal masticatory ability, but this prevention requires strong adherence and regular dental care. Data suggest that expanding coverage of periodontal treatment among patients with T2DM has the potential to prevent tooth loss in more than 30% of the cases (6). Encouraging patients with T2DM and poor oral health conditions to receive periodontal treatment could improve oral health conditions and reduce costs related to the treatment of diabetes and its complications (6). However, numerous interferences limit the patients’ access to treatment, including socioeconomic and educational factors. These factors determine an individual’s behavior and health perception (7).

Another factor that can impact oral health is anxiety. This feeling can be fueled by situations related to dental care, which cause apprehension and discomfort, culminating in avoidance of care and aggravating the oral condition (8). A study reported that anxiety about dental treatment might even impact the quality of life of these patients (9). Only a few studies in the literature have evaluated oral health and the impact of dental anxiety on the use of dental services, diet quality, and glycemic control in patients with diabetes.  The aim of the present study is to evaluate the impact of oral health and the number of teeth on the quality of diet and, consequently, on the glycemic control of patients with T2DM.

## MATERIALS AND METHODS

Cross-sectional study including patients with a previous diagnosis of T2DM, aged 18 years or older, following up at the endocrinology outpatient clinic at a teaching hospital in southern Brazil from August 2017 to July 2018. Patients with cognitive disorders that prevented the understanding of the research proposal were excluded. The sample was chosen randomly among patients seen in the period described and recruited using the electronic medical record system of *Hospital de Clínicas de Porto Alegre* (HCPA).

This study followed the principles of Resolution 466/2012 and was approved by the Research Ethics Committee on Humans of HCPA under CAAE number 70321717.2.0000.5327. All participants received information about the research objectives and procedures and agreed to participate by signing an informed consent form.

The sample size was calculated considering a margin of error of 0.01%, 95% confidence levels, and bilateral correlation coefficient test (r value -0.46), resulting in a calculated sample of 65 individuals with T2DM. The manuscript was prepared according to the Strengthening the Reporting of Observational Studies in Epidemiology (STROBE) recommendations.

### Study variables

To assess diabetes self-care, we applied the Summary of Diabetes Self-Care Activities Measure (SDSCA) questionnaire (10). The questionnaire has 18 questions covering the following domains: general diet, specific diet, exercise, medication taking, blood glucose testing, foot care, and smoking.

To assess the perception of the individuals about their oral health and the impact of oral health on their quality of life, we used the index “chewing ability”, adopted in epidemiological surveys (11). This index consists of five questions about the ability to chew or bite certain types of food and the possible answers were “yes” or “no”. This scale was assigned a score ranging from zero to five, were then classified as having a deficient (score 0 to 3) or satisfactory mastication (score 4 to 5).

For assessment of the participants’ dietary intake, quantitative data were obtained regarding the frequency of food consumption using the Food Frequency Questionnaire – Porto Alegre (FFQ – Porto Alegre) (12) validated for populations of adolescents, adults, and older adults in southern Brazil. The data for the calculations were obtained from the Centesimal Composition of Food table of the Brazilian Institute of Geography and Statistics (IBGE) (13) or from the Brazilian Table of Food Composition (TACO) (14), according to availability.

Data were collected from electronic medical records regarding the following aspects: age, sex, ethnicity, education level, diabetes duration, medications used, and complications associated with diabetes. The social-demographic, and economic profile of the patients were evaluated according to the socioeconomic classification criteria of the Brazilian Association of Research Companies (ABEP) (15). To assess the participant’s anxiety level regarding dental treatment, we used the Corah’s Dental Anxiety Scale (16).

Data related to the individual’s oral health were assessed using the Individual Oral Health Questionnaire 2013 by the National Health Survey (PNS) in partnership with IBGE (17). The original version of the questionnaire consists of 18 items evaluating some oral health variables and we added to the original version two questions related to the receipt of guidance regarding oral health by the dentist or endocrinologist to assess the multidisciplinary interaction between these two specialties.

The dental examination recorded the number and distribution of natural teeth, missing dental groups, presence and identification of the type of dental prosthesis and information related to the alteration of salivary perception and taste sensitivity, xerostomia and presence of cavitated carious lesions. We used the CPO-D Index to evaluate tooth loss. Considering the 28 teeth present in the dental arches (excluding the third molars), the variable “number of teeth” was dichotomized, and individuals with fewer than 21 teeth in the mouth were classified as having “non-functional dentition,” while those with at least 21 teeth were considered to have “functional dentition,” as defined by the World Health Organization (WHO) (18).

The results of glycated hemoglobin (HbA1c) performed up to three months from the date of the interview were collected from electronic medical records. Those without HbA1c measurement during this period had blood collected for this purpose regardless of fasting status. Levels of HbA1c were measured by high-performance liquid chromatography (HPLC). To assess the nutritional status of the study population, the participants’ body mass index (BMI) was calculated based on weight and height data obtained from medical records.

### Statistical analysis

The analysis was performed using SPSS, version 18.0 (IBM, Armonk, NY, USA). Descriptive data with normal distribution were presented as mean and standard deviation, and nonparametric data as median, percentile, or frequency. The Shapiro-Wilk test was applied to analyze the normality of the values referring to the SDSCA items.

Student’s t test was used to compare self-perceived masticatory ability, degree of anxiety about dental care, HbA1c, and diabetes self-care with the variable “number of teeth present”. This test was also used to compare the glycemic control between groups that reported having or not having difficulty in eating and among those with preference for solid or liquid/pureed foods, considering a significance level of 0,05.

The chi-square test was used for categorical variables, while the Spearman test was used to analyze the correlation between the variable “number of teeth present” and self-perceived masticatory ability, degree of anxiety, glycemic control, and items in the T2DM treatment adherence questionnaire and diet quality.

Data regarding the adherence of study participants to the items of the Diabetes Self-Care Activities Measure (SDSCA) questionnaire were expressed as median (interquartile range 25-75) in days per week for self-care activities in the previous 7 days.

## RESULTS

A total of 618 potentially eligible patients were identified from August 2017 to July 2018; of these, 108 were recruited. Overall, 32 individuals did not sign the consent form (mostly due to lack of time to respond to the questionnaires) and 10 were excluded. The reasons for exclusion were failure to attend the scheduled interview (n = 6), questionnaire interruption due to fear of being late for the medical consultation (n = 2), and family members not knowing whether the presence of the subjects in the research could be confirmed (n = 2), yielding a final sample of 66 patients with T2DM.

The final sample comprised adult patients aged 59.7 ± 10.2 years, mostly women (54.5%) and white (66.7%), with a family income of up to 3 minimum wages (54.5%) and complete elementary school education level (44%) (Table 1).

**Table 1:** Clinical and demographic characteristics of the study population – Porto Alegre, RS (n = 66)

Variables		N	% (Mean ± SD)
Sex (% women)		36	54.5
Age (≥50 years)		55	83.3
Ethnicity (% white)		44	66.7
Education (% complete elementary school)		29	44
Family income (up to 3 minimum wages)		36	54.5
Age at diabetes diagnosis (years)			42.9 ± 10.7
Diabetes duration (years)			17.6 ± 9.2
Active or previous smoker		41	62.1
HbA1c ≥8.5 (%)*		35	53
Diabetes complications		57	86.4
	Retinopathy	26	39.4
	Nephropathy	18	27.3
	Neuropathy	13	19.7
Cardiovascular diseases**		36	54.5
BMI (kg/m²)***			32.1 ± 6.5
Normal weight		7	10.7
Overweight or obesity		59	89.3
Use of medications			
	Statins	54	81.8
	Metformin	52	78.8
	Rapid-acting insulin	31	47.0
	Short-acting insulin	56	84.8

* Glycemic control according to the HbA1c levels recommended by the American Diabetes Association for this patient profile.

** Cardiovascular diseases (AMI: acute myocardial infarction; PAD: peripheral arterial disease)

*** BMI: body mass index.

The mean diabetes duration was 17.6 ± 9.2 years, and the mean age at diagnosis was 42.9 ± 10.7 years. As for the occurrence of comorbidities, there was a high prevalence of overweight and obesity, with a mean BMI of 32.1 ± 6.5 kg/m^2^, and a high mean HbA1c, and a high mean HbA1c level (8.9 ± 1.5%), showing poor glycemic control. Complications of diabetes were observed in 86.4% of the participants. About 71% of the patients reported no practice of any type of regular physical activity in the week prior to the interview (Table S1).

**Table S1:** Frequency of diabetes self-care activities in the previous week reported by the study participants – Porto Alegre, RS (n = 66)

Markers	None	1x	2x	3x	4x	5x	6x	7x
	%	%	%	%	%	%	%	%
Healthy diet	15	1.5	5	12	6	18	1.5	41
Professional food guidance	12	0	5	12	13	20	3	35
Blood glucose monitoring	17	11	3	8	3	1	0	57
Physical activity (at least 30 minutes)	71	3	0	5	1.5	3	1.5	15
Specific physical exercise	74	1.5	5	3	1.5	1.5	1.5	12

Specific physical exercise (swimming, cycling, and walking; excludes activities at home or at work).

Regarding the dental conditions of the participants (Table 2), the first tooth loss occurred at an early age (26.9 ± 11.8 years). Only 21.2% of the respondents received information from their dentists about the importance of oral health care in their T2DM treatment, and only 18.2% received information from their endocrinologists about the importance of glycemic control on diseases related to the oral cavity. On clinical examination, frequent use of dental prostheses (65.2%) was verified as a result of the high rate of bilateral tooth loss (93.9%), and the main reason for the last appointment was due to problems related to maladaptation or fracture of the tooth dental prosthesis (37.9%). Most patients reported a frequency of visits to the dentist at intervals of 2 years or more (36.8%) and care center in the private system (60.6%). Given the possibility of delay in seeking dental care due to anxiety or fear concerning dental treatment, moderate to extreme anxiety about dental treatment was observed in 24.3% of the patients.

**Table 2:** Dental characteristics of the study population – Porto Alegre, RS (n = 66)

Variables		N	% (Mean ± SD)
Number of teeth			
	Edentulous	15	22.7
	<21 teeth	34	51.5
	≥21 teeth	17	25.8
Use of dental prosthesis			
	Total	18	27.3
	Removable partial	18	27.3
	Fixed/implant	2	3.0
	Total + removable partial	5	7.6
Missing dental group			
	Incisors	43	65.2
	Canines	33	50.0
	Premolars	60	90.9
	Molar	64	97.0
First tooth loss (years)			26.9 ± 11.8
Bilateral tooth loss		62	93.9
Presence of cavitated carious lesions		21	31.8
Xerostomia		37	56.1
Salivary change		23	34.8
Taste sensitivity		11	16.7
Frequency of oral hygiene			
	Two times or more a day	61	92.5
	Does not use dental floss	51	77.3
Brush change (less than 3 months)		29	44.0
Perception of oral health (bad)		20	30.3
Degree of difficulty in feeding			
	No pain or slight difficulty	44	66.7
	Regular to very intense	22	33.3
Last visit to the dentist (2 years or more)		24	36.8
Preference for some type of food			
	Solid	3	4.5
	Liquid/pureed	27	40.9
Gingival bleeding		17	25.8
Tooth mobility		17	25.8

Although almost all participants (92.5%) reported performing oral hygiene two or more times a day, the use of dental floss was largely neglected. The prevalence of cavity carious lesions was low (31.8%). However, this rate may have been underestimated, since the study was carried out on an outpatient setting without equipment to differentiate carious lesions in terms of activity (active or inactive) and extension (enamel or dentin injury). Other oral manifestations commonly observed in patients with T2DM were detected in the study, namely, xerostomia (56.1%) and changes in salivary appearance (34.8%) and taste sensitivity (16.7%).

Still regarding the dental characteristics of the participants, due to the reduced number of teeth, only a few patients (4.5%) had preference for solid foods. On the other hand, most of them (66.7%) reported mild difficulty in eating or no pain. Most participants (64%) reported consuming fruits and/or vegetables daily (Table S2). A comparison between the groups that reported having versus not having feeding difficulties showed no statistical difference in HbA1c values (9.2 vs. 8.6%, respectively; p = 0.126).

**Table S2:** Frequency of consumption of food items in the previous week, reported by the study participants – Porto Alegre, RS (n = 66)

Items	None	1x	2x	3x	4x	5x	6x	7x
	%	%	%	%	%	%	%	%
Fruits and/or vegetables	5	3	1	9	6	9	3	64
Red meat or foods with whole milk and dairy products	3	9	14	9	8	3	3	51
Sweets	46	29	12	6	0	1	0	6

When the preference for the type of food was evaluated, patients who reported difficulty in eating solid foods had a higher mean HbA1c value than those reporting difficulty in eating liquid/pureed foods, although this result was also not significant (10.2 vs. 9.1%, respectively, p = 0.152). In the correlation analysis, the number of teeth was positively and strongly associated with masticatory ability (p = 0.001; r = 0.44), but a negative correlation was observed between the number of teeth and consumption of five or more servings of fruits and/or vegetables in the previous week (p = 0.001; r = -0.39). These data are shown in Table 3.

**Table 3:** Spearman’s correlation coefficient between the number of teeth and masticatory ability, degree of anxiety, HbA1c level, and items of the Diabetes Self-Care Activities Measure (SDSCA) questionnaire – Porto Alegre, RS (N = 66)

Variables	r	P value
Masticatory ability	0.44**	0.001
Degree of anxiety	- 0.12	0.314
HbA1c	- 0.02	0.827
SDSCA – Follow a healthy diet	- 0.12	0.324
SDSCA – Follow food guidelines	0.06	0.599
SDSCA – Eat five or more servings of fruits and/or vegetables	- 0.39**	0.001
SDSCA – Eat red meat and/or whole milk products	0.05	0.685
SDSCA – Eat sweets	- 0.07	0.565
SDSCA – Perform physical activities for at least 30 minutes	0.01	0.926
SDSCA – Perform specific physical activities (walking, swimming, etc.)	0.02	0.871
SDSCA – Assess blood glucose	0.06	0.603
SDSCA – Assess blood glucose the recommended number of times	0.14	0.260

** The correlation is significant at the 0.01 level.

**Figure 1:**
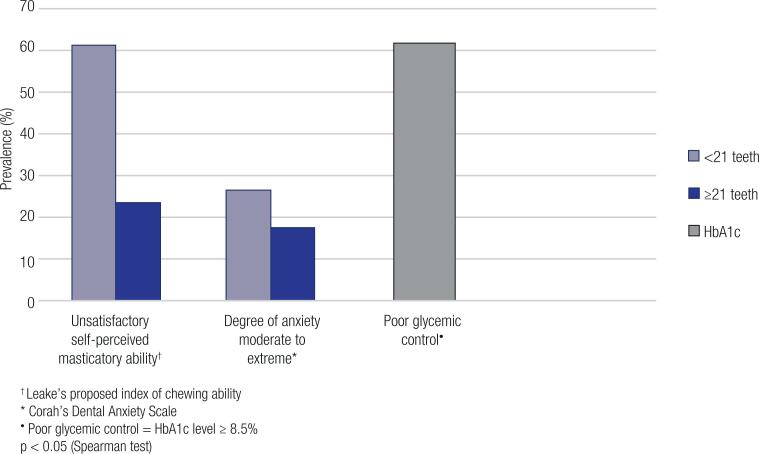
Comparison between groups of patients with fewer and more than 21 teeth – Porto Alegre, RS (N = 66).

Stratified analyses according to the number of teeth, shown in Figure 1, pointed out to a direct relationship between fewer teeth (<21 teeth) and increased prevalence of unsatisfactory self-perceived masticatory ability (p = 0.007). More than half of the patients (51.5%) had an unsatisfactory masticatory ability (Table S3). However, the mean HbA1c between groups with fewer versus more than 21 teeth was similar (8.9 vs. 8.7%, respectively, p = 0.597).

**Table S3:** Distribution of responses on masticatory ability of certain foods – Porto Alegre, RS (n = 66)

Type of food	Index	Yes		No		Total%
		N	%	N	%	
Whole apple with peel, not sliced	5	30	45.4	36	54.6	100
Steak, meat and rib	4	2	3	64	97	100
Raw carrot (whole)	3	4	6	62	94	100
Raw salad	2	29	44	37	56	100
Cooked salad	1	1	1.5	65	98.5	100
None of the above	0	0	0	0	0	0

Score of 0-3: deficient or unsatisfactory masticatory ability; Score of 4-5: satisfactory masticatory ability.

Table S4 shows that the median energy consumption (in kcal/day) of the sample was lower among individuals with fewer than 21 teeth (1949.4 kcal) compared with those with more than 21 teeth (2041.8 kcal), although this difference was not significant (p = 0.73). The SDSCA results showed lower adherence to the items “consumption of sweets” (25th-75th interquartile range [IQR] 0-2), “performing physical activities for at least 30 minutes” (25th-75th IQR 0-3), and “performing specific physical activities” (25th-75th IQR 0-1.2), but greater adherence to items related to “fruit and/or vegetable consumption” in the control of T2DM (25th-75th IQ 4.7-7) in patients with fewer than 21 teeth. The data obtained are shown in Table S5.

**Table S4:** Daily nutrient consumption according to the dental status of the study population – Porto Alegre, RS (N = 66)

Daily nutrient consumption	<21 teeth (median)	≥21 teeth (median)	P value
Energy (kcal)	1949.4	2041.8	0.73
Protein (g)	92.0	99.9	0.90
Lipids (g)	53.6	71.5	0.83
Carbohydrate (g)	271.8	255.7	0.63
Fiber (g)	34.8	29.2	0.50
Iron (mg)	14.8	14.1	0.87
Sodium (mg)	2030.4	1742.5	0.49
Vitamin C (mg)	182.6	199.2	0.57
Saturated fat (g)	19.6	24.7	0.98
Monounsaturated fat (g)	18.9	23.6	0.75
Polyunsaturated fat (g)	9.2	11.6	0.77
Trans fat (g)	1.8	2.3	0.97

Significant values (p ≤ 0.05).

**Table S5:** Adherence of the study participants to the Diabetes Self-Care Activities Measure (SDSCA) questionnaire items – Porto Alegre, RS (n = 66)

SDSCA Items	Total	Teeth		P value
		<21 (%)	≥21 (%)	
1. Follow a healthy diet	5 (3-7)	74.2	25.8	0.53
2. Follow food guidelines	5 (3-7)	74.2	25.8	0.88
3. Eat five or more servings of fruits and/or vegetables	7 (4.7- 7)	74.2	25.8	0.007
4. Eat red meat and/or whole milk products	7 (2-7)	74.2	25.8	0.62
5. Eat sweets	1 (0-2)	74.2	25.8	0.68
6. Perform physical activities for at least 30 minutes	0 (0-3)	74.2	25.8	0.68
7. Perform specific physical activities (walking, swimming)	0 (0-1.2)	74.2	25.8	0.92
8. Assess blood glucose	7 (1-7)	74.2	25.8	0.34
9. Assess blood glucose the recommended number of times	7 (1-7)	74.2	25.8	0.33

* Data expressed as Median (25th-75th interquartile range) in days per week for self-care activities in the previous 7 days. Significant values (p ≤ 0.05). Data related to the intake of diabetes medications (insulin, pills, and injections) are not presented in the table because all patients reported adhering to this item.

## DISCUSSION

This study aimed to describe the oral health profile perceived by patients with diabetes, evaluate the impact of tooth loss on diet quality and glycemic control, and provide information on the use of primary dental care for patients with T2DM treated at a teaching hospital in southern Brazil. A very low prevalence of dental care was identified among the patients with T2DM evaluated, despite a high prevalence of missing teeth and unsatisfactory chewing ability.

Most participants had low education level and family income, a finding similar to other studies. According to the authors, the lower the educational level, the greater the dissatisfaction with chewing and the greater the prevalence of negative impact on oral health, lifestyle, access to services, and information on health care (20).

Despite available care offered by the Unified Health System (SUS), access to dental services in our study (characterized by the dental care report) was 60% in the private system. Oral health care is still considered an important challenge for the SUS network (21), despite the availability of several specific programs (22). This is reflected in lower participation of dentists in the public health care system, and access to this care often remains conditioned on out-of-pocket payment. In this study, the age of the patient when the first tooth loss occurred was quite early (26.9 ± 11.8 years), and about a third of the sample had their last visit to the dentist 2 years or more before the study interview. Prevention of tooth loss, especially at a premature age, requires frequent visits to the dentist and greater awareness by the patient regarding the necessary care to maintain oral health. On average, the patients lost teeth before receiving a diagnosis of T2DM, so other factors seem to have been associated with tooth loss. Social inequalities in the occurrence of oral problems related to tooth loss have been identified in Brazil (23). Some authors state that this type of inequality profile may be due to inequities related to access to dental services, increasing the prevalence of oral problems (24).

From the results found in the present study, there seems to be little concern about oral health among the participants and lack of information on the importance and impact of oral hygiene care on the quality of life and general health of these patients. These findings call for better integration between endocrinologists and dentists in guiding patients with T2DM on the need to maintain adequate glycemic control and oral hygiene in order to minimize the associated risks and increase therapeutic success (25). Patients with T2DM without adequate control have multiple associated oral manifestations (6). Thus, this population was expected to have more regular visits to the dentist and at shorter intervals. Sousa and cols. (26) stressed the need for improved dialogue between dentistry and medicine, pointing out to an approach focused on the principles of integrality.

Tooth brushing along with regular and correct flossing promote effective control of the supragingival dental biofilm and, consequently, interproximal caries and gingivitis (27). However, the introduction of these measures does not significantly improve the indices of plaque and gingivitis if the individual does not know how to perform these tasks satisfactorily (28). As noted by other authors (27), daily tooth brushing was well accepted in our study, but few individuals used dental floss regularly. The prevalence of edentulism in our sample (22.7%) was similar to that found by Huang and cols. in 2013 (26%) (29), but the prevalence of cavitated carious lesions was low (31.8%). This finding may be related to the method used in the study to evaluate carious disease, *i.e.*, performed on an outpatient setting and without equipment to allow for the differentiation of caries lesions in terms of activity (active or inactive) and extension (enamel or dentin injury).

Most participants had fewer than 21 teeth and bilateral tooth loss, which became evident mainly during later dental follow-up evaluations. Tooth loss may be more common in adults with T2DM (6) due to increased exposure to periodontal disease, the most important oral complication and sixth in prevalence among all classic complications of diabetes mellitus (30). According to the literature, these data are strongly associated with the degree of glycemic control, diabetes duration, patient’s age, and presence of associated medical complications (31).

Despite being a self-reported item, masticatory ability was reported as unsatisfactory by more than half of the sample (51.5%). Also, the prevalence of poor masticatory ability was higher than that reported by Figueiredo and cols. (32). Our results showed that the group of patients reporting difficulty in eating solid foods tended to have a higher mean HbA1c level than the group of patients with difficulty in eating liquid or pureed foods, although this result did not reach statistical significance (10.2 vs. 9.1%, respectively, p = 0.152). Considering that the mean HbA1c difference between the groups was about 1%, the lack of association may have been due to a lack of statistical power. About 40% of the patients evaluated had a preference for eating liquid or pureed foods. Although these data vary between studies, they are somewhat concerning, as difficulty or dissatisfaction with chewing can lead to dietary restrictions and, consequently, interfere with glycemic control, causing a negative impact on the quality of life of an individual (3). In the current study, the mean HbA1c between the groups with fewer versus more than 21 teeth was similar (8.9 vs. 8.7%, respectively, p = 0.597), and it is not possible to establish a relationship between tooth loss and diabetes metabolic control. The cross-sectional design and the reduced sample size may have limited the identification of these results. However, in a population-based study conducted in Germany, poorly controlled diabetes was associated with an average increase in periodontal attachment loss and increased risk of future tooth loss compared with normal glycemic control (33).

In our sample, half of the patients with T2DM (53%) were outside the goals of glycemic control proposed by the Brazilian Diabetes Society (3) and most patients (86.4%) were at risk for metabolic complications associated with diabetes. The high prevalence of physical inactivity (71%) found in our study is noteworthy. Due to the benefits attributed to the practice of physical exercise, such as improvement in nutritional status, insulin sensitivity, and glucose tolerance favoring glycemic control, physical activity should be encouraged in patients with T2DM (2).

In a recent study that evaluated the relationship between oral health, insulin resistance and resistance training in rats, it was observed that periodontal disease promoted a decrease in insulin sensitivity, due to the release of inflammatory mediators, such as the tumor necrosis factor-α (TNF -α). However, resistance training promoted an improvement in insulin sensitivity in rats with periodontal disease (34). Several authors have already established this bidirectional relationship between diabetes and periodontal diseases. At the same time that the systemic complications of T2DM promote changes in periodontal conditions (35), glycemic homeostasis can be affected by periodontal disease (35,36), as they increase the inflammatory cytokines responsible for insulin resistance (37). In addition, periodontal treatment has been shown to reduce TNF-α levels and improve glycemic control in patients with T2DM (38).

With regard to the consumption of foods considered unhealthy and, therefore, to be consumed at the most once a week, we observed a high frequency of adequate consumption of sweet foods, despite the inadequate metabolic control observed in our patients. Almost half of the sample (46%) had not consumed sweets in the previous week before the interview. Additionally, we observed frequent consumption in the previous week of fruits and vegetables – foods that are considered indispensable for a healthy diet pattern (4). These findings differ from data reported by Kobayashi and cols. (39) and Lima and cols. (40), who demonstrated an inadequate diet based on excessive consumption of these foods.

Despite the importance of these results, we must emphasize that this study has some limitations. The design has limitations inherent to cross-sectional studies resulting from atemporal monitoring. The sample was selected from patients receiving care at a single center and with a low socioeconomic level. The instrument used to collect dietary data (FFQ) also has limitations, since it relies on the respondent’s memory and does not provide information regarding the type of fibers and processing and consistency of foods, which are important factors interfering in the glycemic index. However, studies like the present one investigating the impact of tooth loss on the quality of diet and glycemic control in patients with T2DM are scarce in the literature and contribute to identifying the oral health profile of these patients and promoting strategies to prevent complications and promote health.

Although recommendations for primary treatment of T2DM include attention to oral health, the percentage of patients receiving dental care was low. When treatment was required, the patients often sought private care. The prevalence of tooth loss in patients with T2DM was high and oral changes were frequent, suggesting a need for greater promotion of oral health care and more effective dialogue between dentists and endocrinologists. In conclusion, the findings of the present study indicate that in patients with T2DM it is essential to promote oral health care through regular visits to the dentist and frequent examinations for prevention of tooth loss, increase awareness to the impact of oral health conditions on quality of life and general health, and improve the connection between diabetes and dental care services within the public and private care networks.
